# Role of vaccines in competitive displacement between SARS-CoV-2 viruses as revealed by the modeling of surveillance data

**DOI:** 10.1007/s15010-025-02586-w

**Published:** 2025-08-01

**Authors:** Hani E. J. Kaba, Nikita Srivastava, Felix Hartkopf, Maike Hohberg, Josué A. Bucio-Garcia, Martin Misailovski, Franz-Christoph Bange, Michael Kleines, Tim Friede, Tim Eckmanns, Simone Scheithauer

**Affiliations:** 1 Department of Infection Control and Infectious Diseases (IH&I), University Medical Center Göttingen (UMG), Göttingen, Germany; 2 Robert Koch Institute (RKI), Berlin, Germany; 3 Department of Medical Statistics, University Medical Center Göttingen (UMG), Göttingen, Germany; 4 Institute for Medical Microbiology and Hospital Epidemiology, Hannover Medical School (MHH), Hannover, Germany; 5 Laboratory Diagnostic Center, University Hospital RWTH Aachen, Aachen, Germany

**Keywords:** SARS-COV-2, Vaccines, Surveillance, Mixed models

## Abstract

**Purpose:**

SARS-CoV-2 Omicron onset resulted in the rapid displacement of Delta and subsequent intra-Omicron displacement-events. Evidence of geographical diversity on the dynamics is present between and within countries. Considering Omicron’s immune-evasive potential compared to earlier variants, we investigated whether vaccines have influenced local dominance patterns in Germany.

**Methods:**

We used weekly-binned genomic surveillance-data representing 48 regional outbreaks with as many displacement-events. Displacement occurred when a given variant constituted ≥ 50% of all isolates. We ran mixed-effect models to quantify vaccine-density effects on the time-to-displacement (ttd) and survival analysis to compare displacement-free time-intervals. Finally, we compared antigenic properties between variants, analyzing receptor-binding domain (RBD) epitope mutations and estimating RBD-residues antigenicity in solved S-protein structures.

**Results:**

Analyzing 895,000 isolates, we calculated ttd-intervals for the Omicron-Delta, BA.2-BA.1 and BA.5-BA.2 displacements. Mixed-effect models suggested the more vaccines have been administered, the sooner a displacement occurred, observing stronger effects for vaccines administered in temporal proximity to displacement-events (2–7% shorter intervals for one vaccination per 10 immunity-events). Survival analysis suggested stronger effects for Omicron-Delta than intra-Omicron displacements. In silico analysis of ca. 500 epitopes and 27,000 residues suggested antigenic change-driven selection as a likely explanation.

**Conclusion:**

This is the first indication of vaccines influencing competitive displacement between SARS-CoV-2 species within a single, socio-demographically homogenous healthcare-system. Since Omicron infections resulted in less adverse outcomes than Delta, our results help understanding the success of vaccines in avoiding severe outcomes at population level. This is particularly important for future pandemics with impacts on timeliness and intensity of vaccination campaigns.

**Supplementary Information:**

The online version contains supplementary material available at 10.1007/s15010-025-02586-w.

## Introduction

SARS-CoV-2 has evolved leading to species with different properties. So-called variants of concern (Delta or Omicron), were linked to distinct waves that differed in onset time, speed or duration, especially at regional level [1]. Such waves are characterized by a displacement, where a successor variant displaces a predecessor as the dominant species.

Until mid-2022, the German epidemic was roughly divided into six waves. Thereby, the transitions from the 4th wave to the 5th (Delta to Omicron BA.1/BA.2) and from the 5th to the 6th (BA.2 to BA.5) are of particular interest, due to the unprecedented rise in reported SARS-CoV-2 cases on one hand, and the accumulation of mutations in the genomes of involved species on the other. These transitions occurred a year after the German vaccination campaign had begun.

Competitive displacement between species of ecological homology, arising from competition for habitat space, is a common phenomenon in nature, involving plants, animals, and microbes, including viruses. The main reasons lie in different fitness properties, dependent on the genomes of competing species. However, it is intuitive that not only virus-related properties alone influence displacement dynamics, since a variety of factors (e.g., pharmaceutical or other interventions) occur and interact with virus properties, favoring distinct entities over others in terms of fitness. For example, reports of potential extinction of the influenza B/Yamagata lineage (not detected since March 2020), have been linked to the recent SARS-CoV-2-pandemic, while other respiratory viruses remain evident [2]. As Omicron had higher odds to infect vaccinated individuals compared to Delta [3], we wondered whether vaccines have influenced displacement-dynamics between the different variations of SARS-CoV-2 at population level. While this is a complex undertaking, simplifying an epidemic at national level could help understanding these phenomena in a better way. Since different healthcare, demographic, and socio-economic factors would influence such relationships, any causality could be best studied within a homogenous healthcare system in one country, as evidence of geographical heterogeneities on transmission dynamics between SARS-CoV-2 variants already exist [1].

Our goal was to quantify the effect of vaccination density on the speed of dominance shifts (time to displacement) between SARS-CoV-2 variants during the pandemic, taking Germany as an example. Using modelling of surveillance data, survival time analysis, epitope mapping and in silico antigenicity determination, we describe the influence of SARS-CoV-2 vaccines on the competitive exclusion between SARS-CoV-2 variants.

## Methods

### Data source and processing

We used genomic surveillance data (1/1/2021–30/1/2023), binned by calendar week and stratified to 16 federal states (Table 1). The data derived from the German Electronic Sequencing Data Hub, or were sequenced at the Robert-Koch Institute, complemented by German GISAID-data. The genomic data is available at GitHub and updated weekly [4–6]. Duplicates/ triplicates were removed in addition to sequences with an N content of ≥ 95% and weeks with *n* < 10 reported sequences. Zip-codes (PLZ) of responsible health authorities were extracted from epidemiological data and used for the geographical allocation of sequences to the respective state. When not available, zip-codes of primary diagnostic laboratories were used instead. For GISAID-sequences (EPI_SET ID: EPI_SET_241004xd, 10.55876/gis8.241004xd), already existing geographical information was used for allocation.

**Table 1:** German federal States under observation in this study. The federal States under observation in this study in addition to the absolute number of sequenced virus isolates (n_sequences) and state-wise reported sequencing rates per 100k PCR-confirmed cases and week (sequencing_rate), both in the time-interval between observed (empirical) onset of Delta (em_delta_) and the start of BA.5 dominance (tp_BA.5_)

BL (state)	alternative name in English^a^	n_sequences	sequencing_rate
Baden-Württemberg		224,109	92
Bayern	Bavaria	165,856	47
Berlin		20,240	35
Brandenburg		23,611	52
Bremen		2,956	27
Hamburg		25,626	71
Hessen	Hesse	22,773	18
Mecklenburg-Vorpommern		424	2
Niedersachsen	Lower Saxony	27,031	19
Nordrhein-Westfalen	North Rhine-Westphalia	211,062	57
Rheinland-Pfalz	Rhineland-Palatinate	22,914	34
Saarland		5,676	30
Sachsen	Saxony	91,540	111
Sachsen-Anhalt	Saxony-Anhalt	13,206	34
Schleswig-Holstein		26,558	59
Thüringen	Thuringia	10,926	29
Total (Germany)		894,508	

^a^ According to https://www.bundesrat.de/EN/organisation-en/laender-en/laender-en-node.html

Virus-entities were grouped into four main classes (consult Supplementary Information and Fig. S1). Allocations of single lineages to the classes are presented in data_file_1. Frequencies and proportions of each virus-entity were determined at state and national level, given as a four-weeks rolling proportion (equation Eq. 1).

1 $$\eqalign{& proportion\,of\,virus - entity\,A\,at\,week\,x \cr& = \,{{\sum _{(i = x - 3)}^xfrequency\,i\,of\,A} \over {\sum _{(i = x - 3)}^xsequenced\,isolates\,i}} \cr} $$

All data processing steps were done using Python. For the management of dates, consult the Supplementary Information. Figures were prepared using Microsoft PowerPoint.

### Temporal variables

For constructing variables, at least one of the following time-points was required:

- Empirical emergence (em): the date a virus-entity was first reported in a given state.
- Estimated emergence (ee): the first day of the month in which the same entity was (nationally) reported first. It assumes the presence of an entity in all federal states once detected in one state.
- Tipping point (tp): the first date when a given entity constituted ≥ 50% of the total pool.

### Outcome

The outcome was the time-interval needed to achieve dominance (≥ 50%), for short ‘time-to-displacement’ (ttd), given in two proxy variables: observed and estimated ttd (Eqs. 2–3).

2 $$\left( {{\rm{observed}}} \right)\,{\rm{:}}\,{\rm{tt}}{{\rm{d}}_{{\rm{obs}}}}{\rm{ = tp - em}}$$

3 $$\left( {{\rm{estimated}}} \right)\,{\rm{:}}\,{\rm{tt}}{{\rm{d}}_{{\rm{est}}}}{\rm{ = tp - ee}}$$

**Fig. 1:**
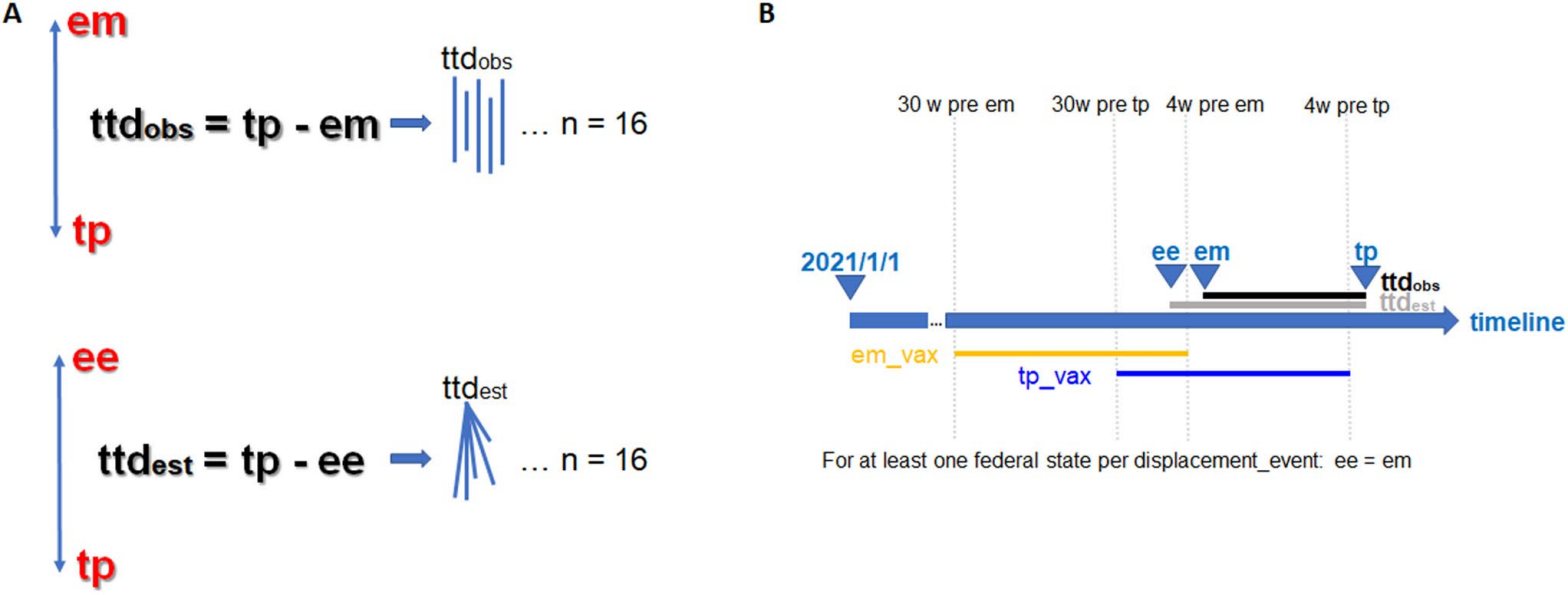
**A** Schematic description of the ttd outcome proxies ttdobs and ttdest. **B** Schematic description of the observation intervals defined to calculate the vaccine density proxies, em_vax and tp_vax

For a given displacement, state-wise ttd_est_-intervals started at the same time-point, while ttd_obs_-intervals had individual starting time-points, being thus prone to bias due to delayed reporting, in contrast to ttd_est_ (Fig. 1A).

### Definition of predictors

The main predictor was vaccination-density before displacement. We pondered which time lags would be sufficient to observe an effect of vaccinations, since it is currently unclear what cut-off the levels could be considered as ‘beneficial’ regarding the extent and duration of protection. A previous German study found that antibody levels as well as avidity in immunologically naïve people peaked about four weeks after the first vaccination on average [7], with similar observation made after infection [8]. We choose four weeks, because at that time, most vaccinated individuals will expectedly have high-avidity antibodies at high levels, while the influence on future outcomes may begin just two weeks after infection/vaccination. Next, we considered the time-interval expected for immunity waning. After infection with Coronaviruses, individuals may have a stable infection protection for at least six months, dependent on a number of factors including HLA-constellation [9]. The effect is not expected to be significantly different in the case of vaccinations, depending on the respective variants circulating, including immune-evasion properties respective to the given vaccine. Thus, we observed vaccination data for six months, ending 4 weeks prior to the respective endpoint of interest. We thereby distinguished between two vaccine-density populations (Fig. 1B):

- em_vax: density of vaccine-doses administered per 100 population between 30 and 4 weeks before onset (em) of a successor.
- tp_vax: density of vaccine-doses administered per 100 population between 30 and 4 weeks before displacement (tp), i.e., beyond onset of a successor.

SARS-CoV-2 case-densities (em_cases and tp_cases) were constructed exactly the same as the vaccine-densities respectively. Vaccine and case-densities were used to calculate the vaccine-to-immunity ratio given in Eqs. 4–5.

4 $${\rm{vax\_ratio\_em = em\_vax / }}\left( {{\rm{em\_cases + em\_vax}}} \right)$$

5 $${\rm{vax\_ratio\_tp = tp\_vax / }}\left( {{\rm{tp\_cases + tp\_vax}}} \right)$$

Next, we restricted the analysis to booster doses (usually third doses) administered in the population, and treated them exactly the same as the total vaccine variables above, using the same observation intervals (Eq. 6 and Eq. 7, consult Suppl. Information, section “Considerations on first booster doses (third doses))”.

6 $$\eqalign{& {\rm{boost\_1\_ratio\_em = em\_boost\_1 }} \cr& {\rm{/ }}\left( {{\rm{em\_cases + em\_boost\_1}}} \right) \cr} $$

7 $$\eqalign{& {\rm{boost\_1\_ratio\_tp = tp\_boost\_1 }} \cr& {\rm{/ }}\left( {{\rm{tp\_cases + tp\_boost\_1}}} \right) \cr} $$

Finally, we observed the proportion of the population with a minimum of one vaccine dose (proportion_total_min1) or with basic immunization (proportion_total_basic) at state level. Both variables were observed roughly four weeks before the occurrence of displacement_1 at national level (reporting day 12/12/2021; people with basic immunization were still considered to be fully vaccinated at that time). For a complete information on predictors and other covariates (rate of predecessor decay, delay in notification, and density of genomic sequencing per infection case), consult data_file_2, including information on the sources.

### Statistical analysis

All displacements were pooled assuming independent ttds across states and virus-entities. We fitted multivariable linear mixed-regression models with log-transformed outcome (natural logarithm-function), allowing effects for random intercept (federal states). Thus, we had different measuring times corresponding to displacements, nested in the states (2-level analysis). Any state was measured as many times as displacements occurred. Regression-coefficients of predictors were transformed to linear scale for interpretation.

In order to compare displacement-free intervals between states of low (< median tp_vax within single displacements) and high (≥ median) vaccination-density, we applied survival-time analysis (Kaplan-Meier, log-rank test), subsequent to the onset of Omicron, BA.2 and BA.5 separately, using ttd_est1_ as time-variable. Bivariable correlation between the proportion of vaccinated population and the outcome (ttd_est1_ or ttd_obs1_ of displacement_1) was performed using Spearman’s rank correlation test (coefficient ρ). All statistical analysis was performed using IBM SPSS Statistics 29.

### Spike protein epitope analysis

Experimentally confirmed epitopes of SARS-CoV-2 (ID:2697049) spike glycoprotein (S-protein, [P0DTC2]), exclusively assayed in human host material, were identified using the Immune Epitope Database (IEDB) [10]. For discontinuous B-cell epitopes, the query was performed on 29/2/2024. Non-WT-epitopes were removed according to outbreak.info R-package data in 53 virus entities (Supplementary Information) [11]. Duplicates were removed as were epitopes < 4 residues [12]. However, unlimited number of gap-residues were allowed between epitope-residues. Only receptor-binding domain (RBD)-epitopes were analyzed, since ca. 90% of the plasma or serum neutralizing antibody-activity against SARS-CoV-2 target the RBD, i.e. residues 320–541 [13, 14]. The most frequent mutations occurring in Delta (10 most prevalent lineages), BA.1, BA.2, and BA.5 (≥ 1% prevalence; last four weeks before displacement) were collected from outbreak.info. Following rules were considered: mutation-prevalence ≥ 75% in all GISAID-German isolates, strain-prevalence ≥ 0.1% since the start of the pandemic, mutation present in ≥ 50% of included entities.

The same strategy described by Muik et al. was applied for linear B-cell (minimum peptide length = 5 aa), MHC I-restricted T-cell (peptide length 8–14 aa) and MHC II-restricted T-cell (peptide length 12–20 aa) epitopes separately [15]. The query was performed on 28/2/2024 for T-cell epitopes and on 29/2/2024 for B-cell epitopes. Epitopes were aligned against the sequence of WT-spike 6VXX deposited at the Protein Data Bank (PDB) [16]. Those not matching with the 6VXX-sequence (non-WT) were removed, same as duplicates. The remaining epitopes constituted the final epitope sample of each type. Epitopes harboring at least one mutated residue became “mutated”. For all epitope-types of every virus-pair A and B involved in the same displacement, the proportion of mutant epitopes in one virus among epitopes shared between the other and the WT was calculated as in Eqs. 8–9.

8 $${P_A} = {{{\rm{n}}\left( \matrix{{\rm{WT}}\,{\rm{epitopes}}\,{\rm{mutated}}\,{\rm{in}}\,{\rm{A}}\, \hfill \cr\& \,{\rm{not}}\,{\rm{mutated}}\,{\rm{in}}\,{\rm{B}} \hfill \cr} \right)} \over \matrix{{\rm{n}}\left( {{\rm{all}}\,{\rm{WT}}\,{\rm{epitopes}}} \right){\rm{}} \hfill \cr- {\rm{n}}\left( {{\rm{WT}}\,{\rm{epitopes}}\,{\rm{mutated}}\,{\rm{in}}\,{\rm{B}}} \right) \hfill \cr} }$$

9 $${P_B} = {{{\rm{n}}\left( \matrix{{\rm{WT}}\,{\rm{epitopes}}\,{\rm{mutated}}\,{\rm{in}}\,{\rm{B}} \hfill \cr\& {\rm{}}\,{\rm{not}}\,{\rm{mutated}}\,{\rm{in}}\,{\rm{A}} \hfill \cr} \right)} \over \matrix{{\rm{n}}\left( {{\rm{all}}\,{\rm{WT}}\,{\rm{epitopes}}} \right){\rm{}} \hfill \cr- {\rm{n}}\left( {{\rm{WT}}\,{\rm{epitopes}}\,{\rm{mutated}}\,{\rm{in}}\,{\rm{A}}} \right) \hfill \cr} }$$

95%-Confidence intervals (95%-CI) of proportions were calculated by normal approximation. Test Statistics (*z*) for H_0_: proportion (A) = proportion (B) was determined using the two-sample independent proportions-test (two-tailed, α = 0.05). For complete data-sets, see data_files_3–6.

### Prediction of antigenicity

We retrieved solved S-protein structures (PDB), covering WT, Delta, Omicron (without further specification), BA.1, BA.2, and BA.5 according to respective descriptions. Random sampling was sequentially achieved using the PDB search-window (search terms “SARS-CoV2” and the respective entity) [17], aiming at including > 30 separate chains (11 structures) per entity. Exclusion criteria: non-SARS-CoV-2, predicted structures, local refinements, incomplete S-proteins, complex with ACE2-receptor, nanobodies, and/or non-human antibodies. Included structures were submitted to the DiscoTope-3.0-server using default settings [18]. RBD-status was determined using the RBD-definition above and repeated using predicted domain-definitions (IPRO18548) [19]. B-cell antigenicity of residues was predicted using the variables “calibrated scores” and “epitope” (data_file_7). The Student’s t-test compared means and the χ^2^-test compared proportions (IBM SPSS Statistics 29).

## Results and discussion

### Descriptive analysis

We processed 1,158,952 sequences of 1,111 lineages. Of those, 894,508 (77%) remained in the database after data processing (1,020 lineages).

**Fig. 2:**
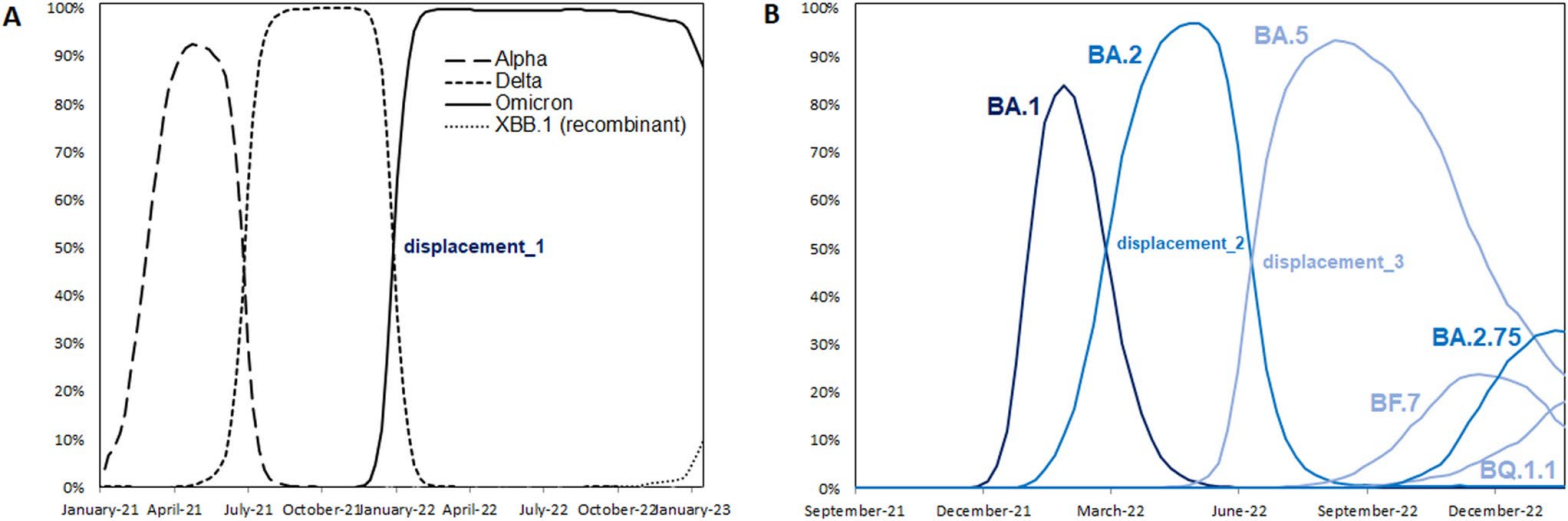
Epidemiological curves of important SARS-CoV-2 entities in Germany based on 894,508 sequenced SARS-CoV-2 isolates. a, Dominant variants of concern between January 2021 and January 2023. b, Omicron clusters between November 2021 and January 2023

Between 1/9/2021 and 30/1/2023, three displacements occurred: displacement_1 of Delta by Omicron, displacement_2 of BA.1 by BA.2 and displacement_3 of BA.2 by BA.5 (Fig. 2). The sequencing rate ranged from 2 (Mecklenburg-Vorpommern) to 111 (Sachsen/Saxony) per 100k PCR-confirmed cases and week between Delta-onset and displacement_3, indicating considerable variability (Table 1). This could potentially lead to delayed reporting in states with low sequencing activity.

**Fig. 3:**
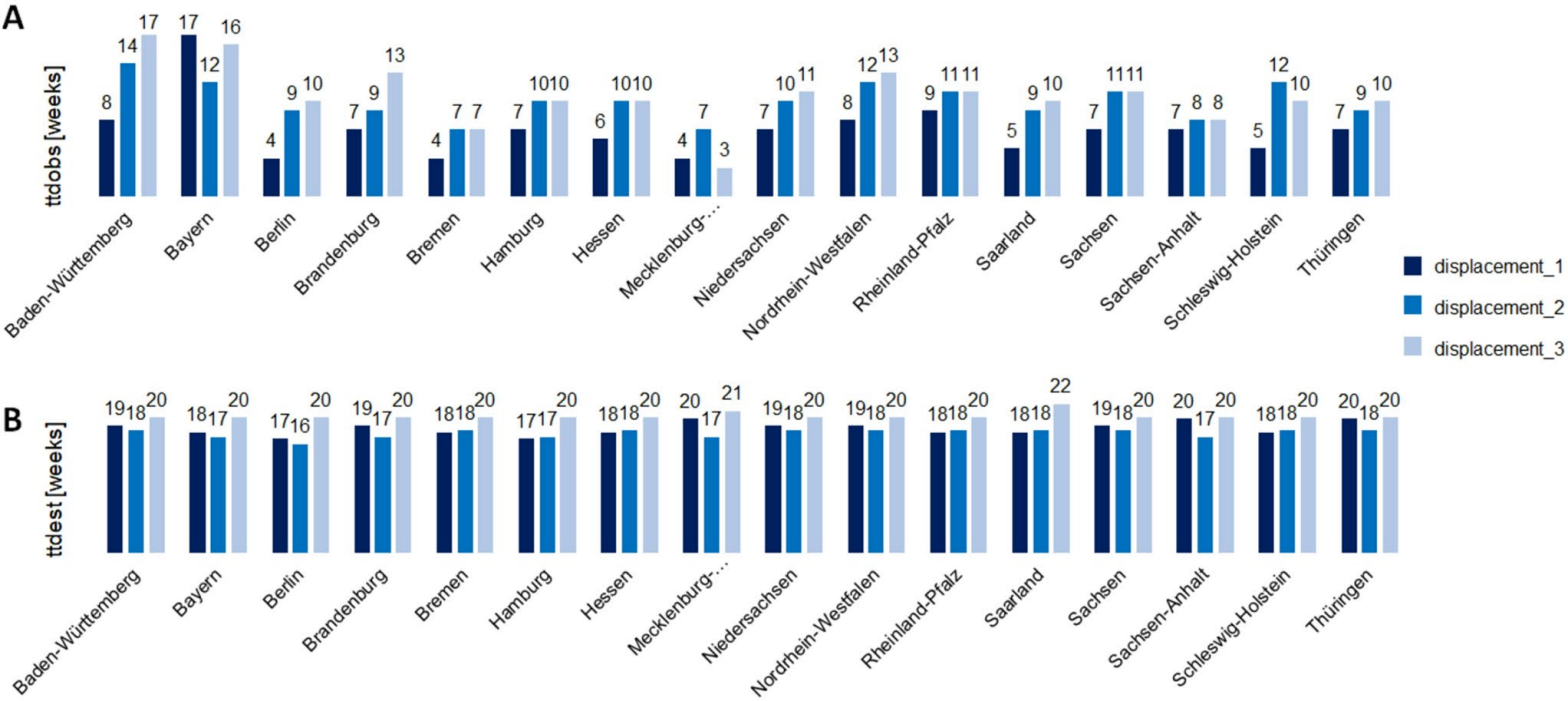
**A** Calculated time-to-displacement values for empirical (ttd_obs_) and **B** for estimated (ttd_est_) across the 16 federal states for the outcome displacement_1 (Omicron dominance), displacement_2 (BA.2 dominance) and displacement_3 (BA.5 dominance)

Figure 3 displays ttd-values across states and displacements. Because ttd_est_ was less sensitive to delays in reporting, it was our main outcome proxy, although it was less variable as compared to ttd_obs_ (SD 1.6 vs. 10.3 weeks).

While Omicron (B.1.1.529) was world-wide first reported in November 2021, our first report dated to 8/9/2021. It took until 1/11/2021 for the next case to be reported, followed by the outbreak. Global GISAID-data even dated the earliest BA.1-sample to 2020 (Supplementary Information Fig. S2) [11]. Whether such information represent misclassifications, contaminations, or cases transiently present in a given country, remains elusive. We considered both onset-dates, resulting in two separate variables for each outcome (ttd_est1_ and ttd_obs1_: reported onset 9/2021; ttd_est2_ and ttd_obs2_: outbreak onset 11/2021).

### Mixed model analysis

Applying mixed-effects linear regression with random intercept (subjects, *n* = 16 states) and repeated measures (*r* = 3 displacements), we modeled log-transformed ttd_est_ by adding the vaccine-to-immunity-ratios of em_vax and tp_vax as predictors, while controlling for sequencing frequencies (tp_seq) and the rate of predecessor-decay (rod). Model M1 (ttd_est1_), suggested that every additional tp_vax administration per 10 immunity events shortened the ttd by 4% on average, while the effect of the temporally more distant em_vax was weaker (Table 2). Repetition for ttd_est2_ (M2) delivered comparable results, as did the modeling of ttd_obs1_ and ttd_obs2_ (SM9 and SM10). Consult Supplementary Information (models SM1–15, Tables S1–4) for detailed documentation of model-variations and Table S5 for details on nonsystematic variance in models M1 and M2.

**Table 2:** Log-linear models with fixed and random effects. Positive effect size values indicate prolongation of the predecessor dominance phase (i.e., slower transition to successor dominance), while negative effect size values indicate reduction of the predecessor dominance phase (i.e., faster transition to successor dominance)

model	Covariate	reg. coefficient	p	95%-CI		vax interpretation [95% CI]
				lower	upper	
M1	Intercept	3.597	0.000	3.362	3.833	
	rod	0.049	0.081	-0.006	0.105	
	tp_seq	-0.207	0.308	-0.619	0.206	
	vax_ratio_em	-0.245	0.145	-0.585	0.095	1 additional vaccine administration per 10 immunity events within 6 months before successor onset → ttd 2% [-1–6%] shorter
	vax_ratio_tp	-0.441	0.004	-0.720	-0.163	1 additional tp_vax administration per 10 immunity events within 6 months before successor dominance → ttd 4% [2–7%] shorter
	Outcome = log_ttd_est1_, restr. -2 log likelihood = -152.4					
M2	Intercept	3.479	0.000	3.284	3.675	
	rod	-0.048	0.112	-0.108	0.012	
	tp_seq	-0.709	0.049	-1.415	-0.002	
	vax_ratio_em	-0.160	0.270	-0.461	0.140	1 additional em_vax administration per 10 immunity events within 6 months before successor onset → ttd 2% [-1–5%] shorter
	vax_ratio_tp	-0.527	0.000	-0.774	-0.280	1 additional tp_vax administration per 10 immunity events within 6 months before successor dominance → ttd 5% [3–7%] shorter
	Outcome = log_ttd_est2_, restr. -2 log likelihood = -91.0					

We concluded: the more vaccines have been administered, the sooner a displacement occurs. The em_vax population showed an effect in the same direction, but with a weaker magnitude and a higher uncertainty compared to tp_vax, probably due to the temporally larger distance to displacements (38–52 vs. 4–30 weeks), in frame of immunity waning [20]. The results also indicate that variable onset dates of Omicron had little influence on the effect.

### Influence of booster doses

Since booster vaccinations (usually third doses) have been demonstrated to enhance effectiveness against Omicron infection and severe outcomes compared to primary vaccination series alone [21], we repeated the analysis by restricting the vaccine variables to third doses only. Running mixed-effects linear regression suggested that every additional tp_boost_1 administration per 10 immunity events shortened ttd_est1_ by 5% on average, while the effect of the temporally more distant em_boost_1 was weaker (Suppl. Information, model SM12). Substituting vax_ratio_tp by boost_1_ratio_tp in model M1 delivered almost identical results (Suppl. Information, model SM13). Repetition for ttd_est2_ delivered again almost identical results (Suppl. Information, models SM14–15). Thus, each vaccine dose acts independently in the same direction, regardless of individual immunity status.

### Correlation of the ttd with population immunity

Finally, we investigated whether the proportion of vaccinated people within the population shows similar associations with the outcome, as single vaccinations did. Indeed, the population proportion with a minimum of one vaccine dose (proportion_total_min1), or with basic immunization (proportion_total_basic), both showed a moderate negative correlation with the outcome ttd_est1_ of displacement_1 (vs. proportion_total_min1: ρ = -0.610, *P* = 0.012; vs. proportion_total_basic ρ = -0.662, *P* = 0.005). Repetition for ttd_obs1_ of displacement_1 delivered comparable results with weaker intensity (vs. proportion_total_min1: ρ = -0.360, *P* = 0.171; vs. proportion_total_basic ρ = -0.342, *P* = 0.194). This would indicate that when more people within the population were vaccinated, the ttd was shorter, consistent with the direction observed for pooled single vaccination data above. While no data on hybrid immunity was available, a previous work suggested that vaccination and natural infection act independently [22]. This is exactly what models M1–2 consider by observing every single immunity producing event.

### Survival time analysis

We pondered whether the obtained results were present in all three displacements alike, given that displacement_1 involved unrelated variants, in contrast to the other two.

We thus compared displacement-free time-intervals between states with low and high tp_vax. The mean difference (Δttd_est1_) was most apparent in displacement_1, in contrast to displacements 2 and 3, indicating that vaccinations acted as suppressors of Delta (Table 3). The cornerstone of this hypothesis is the immune-evasiveness of Omicron in contrast to Delta, being poorly neutralized by antibodies produced by first-generation vaccines or pre-Omicron infection [3, 23]. However, neutralization assays do not return quantifiable epitope differences. Thus, a quantification of antigenic properties was rather needed to investigate the above made assumptions.

**Table 3:** Survival time analysis. Comparison of displacement free time-intervals (ttd_est1_) between States with low (< median tp_vax) and States with high (≥ median tp_vax) vaccine administrations. Analysis was done separately for the displacements 1, 2 and 3 (each *n* = 16)

low_vax (< median) vs. high_vax (≥ median)	mean ttd_est1_ difference	Log rank test
Displacement_1	1.1 [0.3–2.0] 95%-CI	*p* = 0.017
Displacement_2	-0.1 [-0.8–0.6] 95%-CI	*p* = 0.435
Displacement_3	0.4 [-0.2–1.0] 95%-CI	*p* = 0.143

### Immunoinformatic analysis

If vaccinations influence the ttd, they influence different variants with different intensities, dependent on epitope-recognition by the immune system. We quantified epitopes shared by a predecessor and wild-type (WT), but altered in the successor, since all first-generation vaccines were based on WT S-protein.

Usually, > 90% of B-cell epitopes are discontinuous [12]. As 90% of plasma/ serum-neutralizing antibody activity against SARS-CoV-2 target the RBD [13], we observed 74% higher odds for a given RBD-residue belonging to a discontinuous B-cell epitope, compared to non-RBD S-protein residues (OR = 1.74, 95%-CI: 1.69–1.79, *n* = 174k residues from 57 solved structures).

**Fig. 4:**
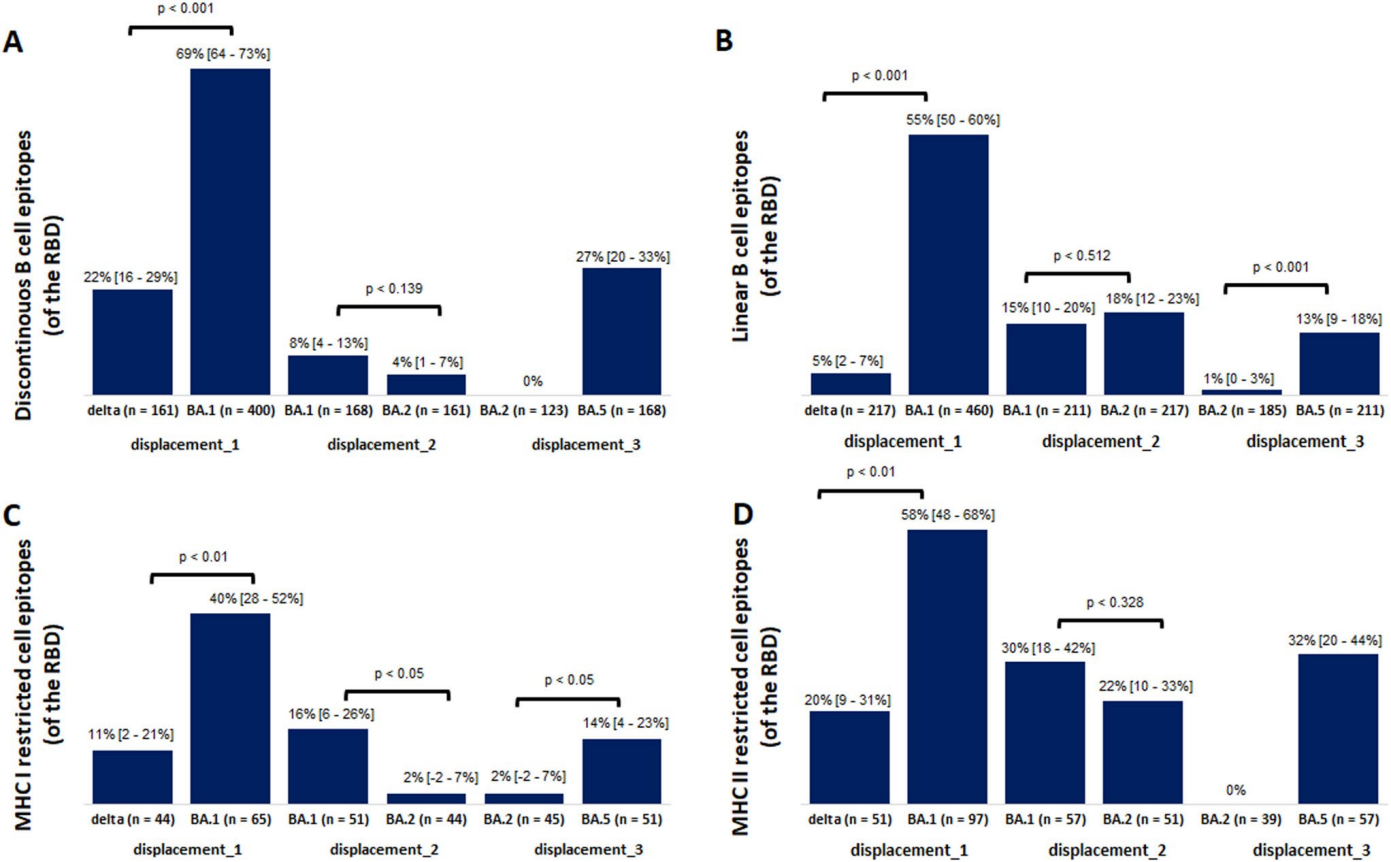
Proportion of receptor binding domain (RBD) epitopes missing in displacement pairs **A** among those shared between B and wild-type and vice versa, for every displacement (1–3). Shown for a, discontinuous B-cell epitopes, **B** linear B-cell epitopes, **C** MHC I-restricted T-cell epitopes and **D** MHC II-restricted T-cell epitopes

We compared predecessor-successor-pairs in 564 discontinuous B-cell RBD-epitopes. BA.1 was the Omicron-proxy (~ 86% of national Omicron reports; last four weeks before displacement_1). BA.1 accumulated more mutations at residues shared between WT and Delta, resulting in a higher loss of WT-epitopes conserved in Delta, than vice versa (Fig. 4A). Similar results were obtained for the other epitope types (Fig. 4B–D). The number of maximal mutation-sites per epitope showed a weak correlation between Delta and BA.1 (*r* = 0.162), since only two potential mutation-sites were present in Delta-RBD, occurring together in 4% (95%-CI: 1–7%) of epitopes, while in BA.1-RBD, 73% (95%-CI: 68–77%) harbored between two and eight mutations at once. These findings support previous results indicating that sera from unvaccinated individuals recovered from BA.1 infection mainly neutralized BA.1 but not pre-Omicron variants and vice versa [23].

BA.1 and BA.2 showed strong correlation of maximal mutation-sites per epitope (*r* = 0.927). The number of mutated epitopes shared between the respective other entity and WT was almost equal for most epitope-types (Fig. 4A–D). Although BA.2 and BA.5 displayed strong correlation of maximal mutation-sites per epitope (*r* = 0.964), the quantifiable epitope landscape was different (Fig. 4A–D), as BA.5 displayed almost no reversed mutations, while obtaining additional mutations absent in BA.2.

**Fig. 5:**
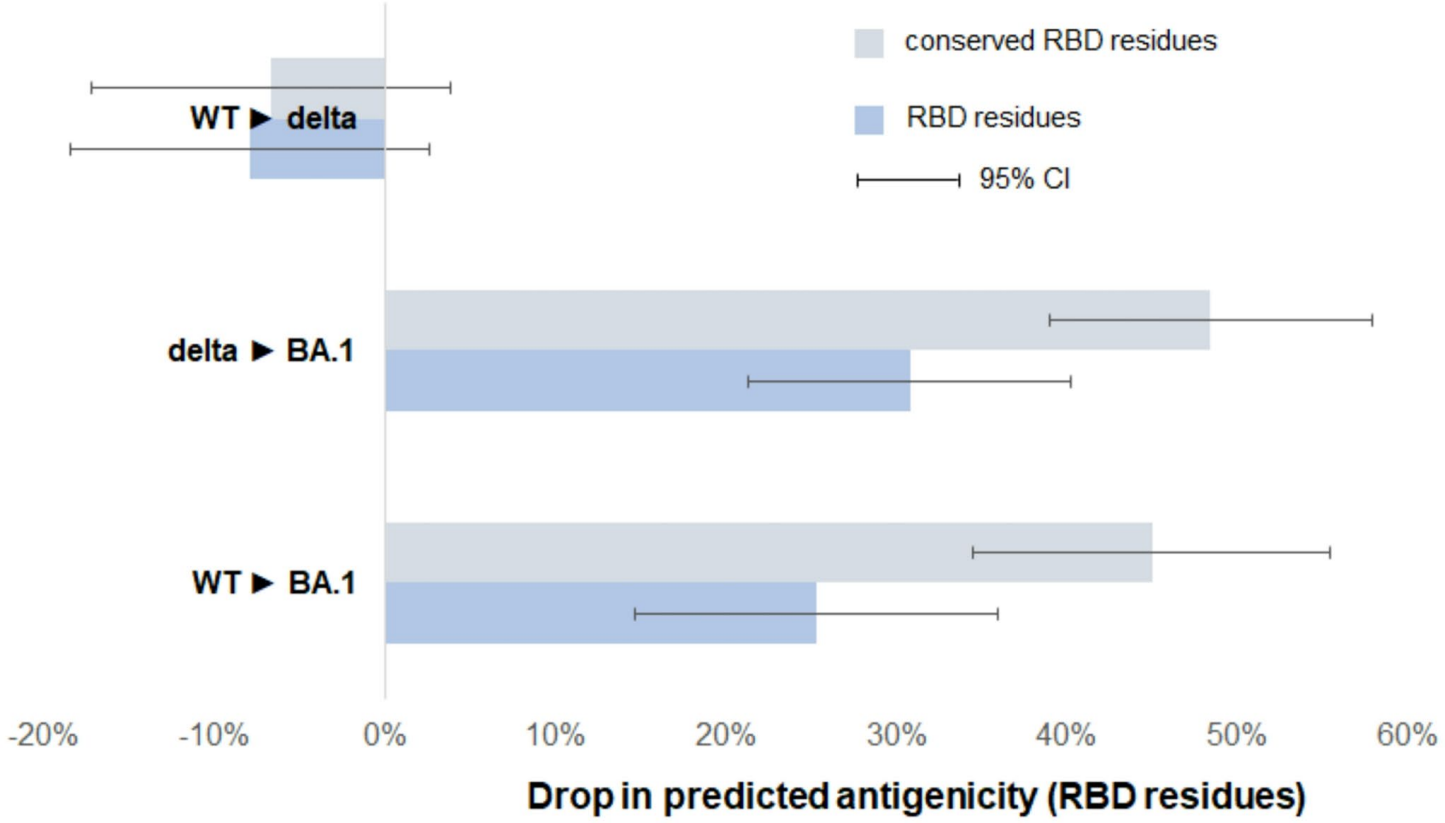
Drop in predicted antigenicity of receptor binding domain (RBD) epitopes per displacement-event, i.e. between different SARS-CoV-2 entities: wildtype (WT, *n* = 6,264 residues), Delta (*n* = 6,808) and Omicron BA.1 (*n* = 6,465)

Using calibrated DiscoTope-3.0 scores, we observed a general drop in antigenicity for BA.1/Omicron compared to both WT (~ 25%) and Delta (~ 30%), also present in BA.1-residues that did not undergo mutation (WT ~ 41%, delta ~ 48%; Fig. 5). The result was reproducible for structures dubbed as “Omicron” (Table 4) and again using alternative RBD-definitions (Suppl. Material, Table S6).

**Table 4:** Antigenicity prediction. DiscoTope-3.0 calibrated scores for receptor binding domain (RBD) residues of SARS-CoV-2 S-protein, using the broad RBD definition (aa 320–541). The higher the calibrated score, the higher the antigenicity of a given residue ^a^

320–541 RBD definition	variant / entity	n (PDB structures)	n (residues)	arithmetic mean	sd
	WT	11	6,264	0.408	1.271
	Delta	11	6,808	0.440	1.229
	Omicron	11	7,317	0.346	1.175
	BA.1	11	6,465	0.304	1.212

^a^ Student’s t-test for comparison between entities: WT–Delta *p* = 0.141, WT–BA.1 *p* < 0.001, Delta–BA.1 *p* < 0.001, WT–Omicron *p* < 0.001, Delta–Omicron *p* < 0.001

Combined, these results suggest lower odds of Omicron for recognition by antibodies directed against the WT, as compared to Delta, supporting previous observations [24]. Expectedly, monoclonal antibodies for SARS-CoV-2-treatment showed lower efficacy against Omicron variants in comparison to WT, than Delta [25]. Since neutralizing antibodies block ACE2-binding in a competitive manner, this would also suggest higher chances of host cell-entry for Omicron as compared to Delta in addition to advantages by reduced recognition [26].

In contrast, the immunogenic similarity in relation to the WT was comparably higher between BA.1 and BA.2 (although distinct differences exist [23]), leading to a diminishing effect of vaccines on any fitness advantages of BA.2 against BA.1, in line with previous results [27]. This is supported by Danish data showing that susceptibility to infection by BA.2 was higher than BA.1 among household contacts, independent of vaccination status. This means other fitness attributes such as inherent increased transmissibility, likely lying behind the advantage of BA.2 over BA.1 [28]. The vaccine-effect on displacement_3 was slightly stronger compared to displacement_2, but much weaker compared to displacement_1, which is supported by epitope analysis. Since BA.5 replicated at higher levels in human nasal epithelium, followed by BA.2 and BA.1, it could be argued that properties other than antigen-driven selection by antibodies had stronger effects on the speed of displacement_3 [29].

### Limitations

The presented results are subject to limitations due to data, model structures, or assumptions. Since hospitalizations or death due to SARS-CoV-2 infection were not relevant for outcome construction, it was not necessary to further stratify vaccinations by vaccine (mRNA-based, vector-based etc.) and/or dose-types (primary, booster, etc.), respective their effectiveness. Previous findings showed that vaccine effectiveness against Delta-transmission was equal between primary and booster vaccinations [30]. However, such results may vary with variable recipient demographics and the time-point of administration. Other limitations derive from the aggregated nature of the data, and the stratification by political borders, which resulted in small numbers of observed units, especially in survival-time analysis. It is important to note that lineage/mutation frequencies could be influenced by total case numbers and the frequency of genomic sequencing at any location and time-interval. Samples submitted for sequencing may not be randomly selected, especially if they derive from single clusters in the context of outbreak investigations, potentially leading to an accumulation of distinct entities that would have otherwise been overlooked [11]. Additionally, a lag exists between the time-point when samples are isolated from patients, when samples were sequenced, and when the resulting data was published/ notified to surveillance systems. This time-lag means that the most recent data often contain fewer samples, potentially limiting timeliness. A recent Swiss study reported a ttd-decrease when fewer sequences were available (by downsampling) in a dose-dependent fashion for a number of variants. This observation was less pronounced for Omicron, as downsampling did not affect its first detection [31]. In our study, this would have been a particular source of concern for states with a comparably low sequencing density and delayed em values. However, higher sequencing events were required for an accurate capturing of variant growth, especially Omicron [31].

On the other hand, our approach has strengths, mainly using different outcome-proxies (observed vs. estimated), onset dates (9/2021 vs. 11/2021), and methods of analysis, including model-structures. All those variations lead to the same conclusions, showing the robustness of our results.

## Conclusions

This is the first evidence for vaccines interfering in competitive displacement between SARS-CoV-2 viruses at population-level, in a socio-demographically homogenous healthcare-system.

We conclude that the more first- generation vaccines were used, the sooner a displacement occurred. This effect was pronounced in the Delta-to-Omicron event (without mandatorily being void in intra-Omicron displacements) and stronger for vaccines administered in temporal proximity to the displacement-event. Our explanation suggests antigenic-change driven selection by antibodies.

The reconstruction of displacement-dynamics is thus important to cope with future developments, in frame of pandemic preparedness. The end of COVID-19 as a public health emergency might have been officially declared, but multiple questions on the course of the pandemic, including its geographical diversity, still require answers. This work closes therefore an important gap towards understanding interactions between pharmaceutical public health interventions and dominance-periods. Part of the success of vaccines in avoiding severe outcomes could be therefore attributed to helping shift dominance towards variants with lower probability of serious adverse outcomes, especially in hospitalized patients [32].

## Electronic supplementary material

Below is the link to the electronic supplementary material.

Supplementary Material 1

## Data Availability

Datasets of variables used in modeling, virus identities, virus frequencies per federal state, RBD epitopes and RBD residue analysis are available online in data_files_1–7, including data dictionaries, accessible via the following Link: https://owncloud.gwdg.de/index.php/s/9AImUp88px6Ki1I. Codes used for virus frequencies (Python), mutation frequencies (R) or modeling (SPSS) are stored in data_file_8, accessible via the same link as above.
