# Supplementary material for: Role of vaccines in competitive displacement between SARS-CoV-2 viruses as revealed by the modeling of surveillance data
Source: Infection. 2025 Aug 1;53(6):2511–21. doi: 10.1007/s15010-025-02586-w (PMC12675727; doi:10.1007/s15010-025-02586-w)

**Role of Vaccines in competitive Displacement between SARS-CoV-2 Viruses as revealed by**

**the modeling of Surveillance Data**

Hani E. J. Kaba^1^*, Nikita Srivastava^1^*, Felix Hartkopf^2^, Maike Hohberg^3^, Josué A. Bucio-Garcia^1^, Martin Misailovski^1^, Franz-Christoph Bange^4^, Michael Kleines^5^, Tim Friede^3^, Tim Eckmanns^2^ and Simone Scheithauer^1^

^1^ Department of Infection Control and Infectious Diseases (IH&I), University Medical Center Göttingen (UMG), Göttingen, Germany

^2^ Robert Koch Institute (RKI), Berlin, Germany

^3^ Department of Medical Statistics, University Medical Center Göttingen (UMG), Göttingen, Germany

^4^ Institute for Medical Microbiology and Hospital Epidemiology, Hannover Medical School (MHH), Hannover, Germany

^5^ Laboratory Diagnostic Center, University Hospital RWTH Aachen, Aachen, Germany

*equal contribution

**Data Availability**

GISAID Identifier: EPI_SET_241004xd

doi: 10.55876/gis8.241004xd

All genome sequences and associated metadata in this dataset are published in GISAID’s EpiCoV database. To view the contributors of each individual sequence with details such as accession number, Virus name, Collection date, Originating Lab and Submitting Lab and the list of Authors, visit 10.55876/gis8.241004xd

**Data Snapshot**

EPI_SET_241004xd is composed of 886,981 individual genome sequences. The collection dates range from 2020-01-28 to 2023-01-27; Data were collected in 1 country and territory; All sequences in this dataset are compared relative to hCoV-19/Wuhan/WIV04/2019 (WIV04), the official reference sequence employed by GISAID (EPI_ISL_402124). Learn more at https://gisaid.org/WIV04.

**Virus entities - Definitions**

Lineage: In the context of this study, (pangolin) lineage refers to a given SARS-CoV-2 strain, that is genetically unique, each designated by a unique identifier following the pangolin nomenclature.

Cluster: Clustering lineages followed two steps. First, all incident lineages in the observation interval were clustered according to the 'pango_alias_list' as of 03 March 2023 (https://github.com/cov-lineages/pango-designation/blob/master/pango_designation/alias_key.json). Allocations of B.1.1.529 descendants were defined according to the first numbers after the prefix in the pangolin designation (e.g., B.1.1.529.1* for BA.1 variant descendants, B.1.1.529.2* for BA.2 variant descendants etc.). In the second step, variants belonging to the most prevalent clusters were treated as independent entities if they were deemed epidemiologically relevant or have been explicitly considered as variant of concern (VOC) or variant of interest (VOI) by the WHO at any time point up to 30 January 2023. Those were: BA.1, BA.2, BA.3, BA.4, BA.5, BA.2.75, BA.4.6, BF.7 and BQ1.1.

Supercluster: A supercluster is a cluster of clusters, descending from a common ancestor lineage (e. g., B.1.1529) that was declared as a VOI or VOC, but became very diverse in later pandemic stages. A supercluster was basically created during the first step of cluster creation (see above). The most prominent examples were the five Omicron superclusters (BA.1 – BA.5).

Variant: Using the same logic as above, superclusters were joined into an entity, dubbed as ‘variant’, such as Omicron, comprising the five superclusters mentioned above. Other variants were Alpha, Beta, Gamma, Delta, Epsilon etc. Variant designations followed the Greek letter nomenclature proposed by the WHO on 31 May 2021. Recombinant entities resulting from ancestral Omicron clusters/ superclusters were treated as independent variants from the given parental Omicron superclusters. Based on those definitions, displacement was understood to possibly occur a) between variants and b) between (super)clusters within a given same variant.

**Management of dates**

Dates were restricted or reduced (if present on daily basis) to weekly data. Following ISO 8601 standards, any week started on Monday and ended on Sunday. The first calendar week of a given year was therefore the week containing the first Thursday of the Gregorian year. In order to avoid data processing errors in terms of calendar weeks, the date of the respective Monday of a given week under observation was thereby used as an unequivocal identifier of that given week. This identifier was used to match virus entity proportions with covariate data according to the equations shown in the main text and below.

**Variability at both ends of ttd_obs_**

On average, local empirical Omicron onset (em) occurred 12 ± 3 [range: 1–13] weeks after the very first reported Omicron onset in Germany, compared to 5 ± 2 [range: 1–8] weeks for BA.2 and 8 ± 4 [range: 1–16] weeks for BA.5 respectively. In contrast, the time-point of tp occurrence was locally less variable (Omicron: 2·5 ± 1·0 [range: 1–4] weeks upon the very first displacement event in Germany; BA.2: 3·0 ± 0·6 [range: 1–3] weeks; BA.5: 1·0 ± 0·5 [range: 1–3] weeks).

**Covariates**

Following covariates were selected

1. em_cases, density of SARS-CoV-2 cases per 100 population between 30 and 4 weeks before the date of onset (em) of a given variant in a given state (in analogy to em_vax).
2. tp_cases, density of SARS-CoV-2 cases per 100 population between 30 and 4 weeks before the date of displacement event (tp) of a given variant in a given state (in analogy to tp_vax).

For covariates 1 and 2, only cases identified by PCR tests were included. Covariates 1 and 2 were treated exactly the same during their construction, as the predictors em_vax and tp_vax respectively.

By construction, tp_cases differed from em_cases by representing a mix of predecessor and successor cases (e. g. Delta and Omicron at the interface between the 4^th^ and 5^th^ wave) rather than predecessor cases only. Omicron cases are correlated with ttd which could result in a low variance, potentially close to zero. Therefore, em_cases was the preferable cases proxy.

1. rod, the rate of decay (per day) of the predecessor (e.g., Delta as predecessor of Omicron, BA.1 of BA.2 and BA.2 of BA.5), defined as the difference in state-wise weekly incidence rates between the time point of maximum proportion in the total pool (max) and the time point of being displaced (tp) by the dominant successor (EQ S.1).

$$EQ S.1: rod=\frac{\mathrm{proportion}\left( \mathrm{predecessor} \right) on tp -max. proportion (predecessor)}{tp-min (date of max. proportion (predecessor))}$$

1. dist, time distance in weeks between the very first report of a given variant in Germany, min(em) and the first report of the same variant in the given state of interest (em; when the prevalence becomes > 0). This variable should account for possible delays in reporting when modelling ttd_obs_, especially in state with a relatively small population, being surplus for modelling ttd_est_. The minimum value of this variable equals 1 week (in order to avoid zero values) according to EQ S.2.

EQ S.2: dist = em – min(em) + 1

1. tp_seq, ratio of genomic sequence results density (after data processing) in the database between 30 and 4 weeks before the date of displacement event (tp) of a given variant in a given state (in analogy to tp_vax), relative to tp_cases. Through this variable we controlled for the density of test results per state. The database consisted of sequence results between em_Delta_ and tp_BA.5_.

**Considerations on mixed effects models**

Data in which there are repeated measurements for each unit of observation, are best analyzed by mixed models (multilevel or hierarchical linear models).

Although random effects are not related to sampling, since we observe all available states, it was necessary to account for potential clustering in the data. A fixed and random effects (i. e. mixed) model with repeated measurements would thus allow us to use all the data at hand, ensuring a higher sample size, and accounting for the correlations between data (residuals), while controlling for fewer parameters for estimations. In the covariance structure, we allowed for heterogeneous variances at the different time points. By using random effects for states, residuals represent the distance between a given data point and the mean for that same state instead of being the distance between a data point and the mean of all states. The measures at the different time points within a given states are however not related to each other because the interval needed for a given successor virus entity to become dominant is independent from the interval needed for its predecessor to become dominant. Therefore, we assumed zero correlation between elements using a Variance Components (diagonal, DIAG) matrix structure (providing separate variance estimates for each random effect, but no covariance between random effects, as in independence models). Furthermore, we preferred the Restricted Maximum Likelihood (REML) method over the Maximum Likelihood (ML), as we had a small number of Level 2 units in a balanced data set.

**Detailed documentation of model variations**

Additional models which include variations to models M1 and M2, are provided in Tables S1–4, Suppl. Material. Model SM1 (ttd_est1_) suggested that every additional up-to-date vaccine administration per 10 population before successor onset (em_vax), prolonged the ttd by 4% on average (95%-CI 2–6%, ceteris paribus; c.p.). However, beyond the emergence of the (evasive) successor, every additional vaccine administration per 10 population (tp_vax) shortened the displacement interval by 4·9% (95%-CI 2·9 – 6·8% on average). These observations remained conserved when controlling for the proportion of sequences among SARS-CoV-2 cases (tp_seq; model SM2) or controlling for a mix of predecessor and successor cases (tp_cases; model SM3) instead of predecessor cases only (em_cases), as successor cases were still outnumbered before any displacement event occurred. Similar results were obtained after modeling ttd_obs1_ (model SM4). Note that em_vax (model SM5) shortened ttd_est1_ but in a weaker manner compared to tp_vax (model SM6), when each vaccine proxy was introduced to the model separately, instead of together, indicating that em_vax was flipping its sign when in the latter case. This could be due to multicollinearity, since em_vax and tp_vax were positively correlated (Supplementary Material Fig. S3 left panel). On the other hand, because tp_vax was temporally closer to the displacement date, it could had a stronger effect (with less inaccuracy) on ttd compared to em_vax. Model SM7 (ttd_est2_) delivered almost identical results to M1. Modeling ttd_obs2_ in presence of tp_vax and em_vax was not possible due to a non-positive final Hessian matrix (model SM8).

Importantly, the predecessor rate of decay (rod) was not significantly associated with the ttd, under the used testing conditions. Thus, the drop in predecessor cases may not have been restricted to fitness disadvantages, independent of one or more external factors. Any fitness advantage of the successor was likely due to an intervention, e.g., the rise of immunity. As immunity can be gained from previous infection (against any circulating virus-entity) or immunization (against a specific entity plus potential cross-immunity), we quantified the share of vaccine administrations (specific immunity) relative to all reported immunity producing events (infections and vaccinations), leading to the main models M1 and M2 presented in the main manuscript text.

One concerning issue of our initial models was the sign flipping behavior of em_vax in SM1 and SM7 once tp_vax was included in the respective model. Theoretically, the outcome (ttd) depends on the immune evasive potential of the successor. If it is high, additional vaccines, administered before successor onset, should not prolong the ttd and vice versa. Same is true for vaccines administered beyond successor onset. So, we would expect both vaccine proxies to have an effect in the same direction. But this is not what we have observed in SM1 and SM7. One explanation could be a strong collinearity between em_vax and tp_vax since they partly overlap, evident by graphical plotting (Supplementary Material Fig. S3, left panel), that is reduced when vaccine counts were normalized by immunity producing events (Supplementary Material Fig. S3, right panel), instead of normalizing by the population (constant for a given federal state). On the other hand, a negative sign of em_vax could also be epidemiologically explained. Before successor onset, vaccines have no effect on the dominance interval of the predecessor, which is circulating independent of vaccinations. Increased vaccine incidence would coincide with infections by the predecessor, which produce secondary cases and thus prolong the dominance interval of the predecessor, thus reducing the ttd of the successor *per se*, creating a typical confounding triangle. Controlling for infection cases might not be sufficient to “fix” this problem, since they do not include secondary cases produced at a later time-point. Once the evasive successor has emerged, a portion of the same em_vax vaccines act as a suppressor of predecessor circulation, leading to gradual reduction of the ttd, but the majority of vaccines have been administered before the successor onset, and have a stronger impact on the ttd. Whether the sign-flipping phenomenon represents an epidemiologically relevant effect, remains therefore elusive. However, the best remedy for the sign-flipping problem was apparently normalizing the vaccine proxies by immunity producing events instead of by the population (per capita).

**Considerations on first booster doses (third doses)**

The 3^rd^ dose was dependent on the variable “Impfserie” in the data source file (https://github.com/robert-koch-institut/COVID-19-Impfungen_in_Deutschland/blob/main/Deutschland_Bundeslaender_COVID-19-Impfungen.csv). In principle, two vaccine doses were required for primary immunization (except for the kids vaccine, until January 2022), and thus 3 vaccine doses should count as a booster in theory. However, it was not always possible to map individual vaccination histories with the data, as only aggregated data were available from physicians' clinics. Ultimately, the “Impfserie” (vaccination series) is reported as it appeared in the data. It should also be noted that at the beginning of the vaccination campaign, a single vaccination was sufficient for primary immunization for individuals with a previous SARS-CoV-2 infection (this first vaccination was to be reported with vaccination series = 2, and a follow-up vaccination with vaccination series = 3). Thus, regarding the 3^rd^ vaccine dose as booster has some limitations. For this reason, we decided to quantitatively observe all vaccine doses administered during the observation interval individually.

**List of virus entities for mutational mapping**

"B.1.1.7*", "B.1.351*", "B.1.617.2*", "B.1.526*", "B.1.525*", "B.1.427*","B.1.429*", "B.1.617.1*", "B.1.621*", "C.37*", "P.1*", "P.2*", "P.3*", "A.23.1*", "A.27*", "B.1.1.318*", "C.36.3*", "XBB.1.5*", "XBB.1.16*", "EG.5*", "CH.1.1*", "BA.2.86*", "BA.2.75*", "XBB.1.9.1*", "XBB.1.9.2*", "XBB.1.5*", "XBB.1.16*", "CH.1.1*", "BA.1.1", "BA.1", "BA.1.18", "BA.1.1.1", "BA.1.17.2", "BA.1.17", "BA.1.15", "BA.2", "BA.2.9", "BA.2.3", "BA.2.36", "BA.2.12.1", "BN.1.3", "BF.7", "BQ.1.1", "BA.5.2", "BA.5.2.1", "BQ.1", "BA.5.1", "BF.7.5", "BA.5.2.6", "BF.14","B.1.177","BA.4*", and "BA.3*".

**Supplementary material Tables**

Supplementary material Table S1. Additional ttd_est1_ or ttd_obs1_ models.

| model | covariate | reg. coefficient | p | 95% CI | |
| --- | --- | --- | --- | --- | --- |
|  |  |  |  | lower | upper |
| SM1 | intercept | 2.946 | 0.000 | 2.781 | 3.110 |
|  | rod | 0.001 | 0.970 | -0.046 | 0.048 |
|  | em_cases | 0.005 | 0.000 | 0.003 | 0.007 |
|  | em_vax | 0.004 | 0.000 | 0.002 | 0.006 |
|  | tp_vax | -0.005 | 0.000 | -0.007 | -0.003 |
|  | outcome = log_ttd_est1_ | |  |  |  |
| SM2 | intercept | 2.930 | 0.000 | 2.774 | 3.085 |
|  | rod | 0.001 | 0.947 | -0.041 | 0.044 |
|  | em_cases | 0.004 | 0.000 | 0.002 | 0.006 |
|  | em_vax | 0.004 | 0.000 | 0.003 | 0.006 |
|  | tp_vax | -0.005 | 0.000 | -0.007 | -0.003 |
|  | tp_seq | -0.706 | 0.001 | -1.119 | -0.292 |
|  | outcome = log_ttd_est1_ | | | | |
| SM3 | intercept | 2.895 | 0.000 | 2.712 | 3.078 |
|  | rod | 0.004 | 0.860 | -0.044 | 0.053 |
|  | tp_cases | 0.005 | 0.000 | 0.003 | 0.007 |
|  | em_vax | 0.003 | 0.002 | 0.001 | 0.005 |
|  | tp_vax | -0.004 | 0.002 | -0.006 | -0.002 |
|  | outcome = log_ttd_est1_ | | | | |
| SM4 | intercept | 3.075 | 0.000 | 2.546 | 3.604 |
|  | dist | -0.083 | 0.000 | -0.096 | -0.071 |
|  | rod | 0.132 | 0.048 | 0.002 | 0.263 |
|  | em_cases | 0.008 | 0.138 | -0.003 | 0.018 |
|  | em_vax | 0.011 | 0.001 | 0.005 | 0.017 |
|  | tp_vax | -0.014 | 0.001 | -0.023 | -0.006 |
|  | outcome = log_ttd_obs1_ | | | | |

Supplementary material Table S2. Additional ttd_est_ or ttd_obs_ models.

| model | covariate | reg. coefficient | p | 95% CI | |
| --- | --- | --- | --- | --- | --- |
|  |  |  |  | lower | upper |
| SM5 | intercept | 2.944 | 0.000 | 2.779 | 3.110 |
|  | rod | 0.031 | 0.259 | -0.024 | 0.086 |
|  | em_cases | 0.008 | 0.000 | 0.005 | 0.010 |
|  | em_vax | 0.000 | 0.577 | -0.002 | 0.001 |
|  | outcome = log_ttd_est1_ | | | | |
| SM6 | intercept | 3.130 | 0.000 | 2.955 | 3.306 |
|  | rod | 0.042 | 0.100 | -0.009 | 0.093 |
|  | em_cases | 0.006 | 0.000 | 0.003 | 0.008 |
|  | tp_vax | -0.002 | 0.013 | -0.004 | -0.001 |
|  | outcome = log_ttd_est1_ | | | | |
| SM7 | Intercept | 2.823 | 0.000 | 2.646 | 2.999 |
|  | Rod | -0.022 | 0.429 | -0.080 | 0.036 |
|  | em_cases | 0.005 | 0.000 | 0.003 | 0.007 |
|  | em_vax | 0.005 | 0.000 | 0.003 | 0.007 |
|  | tp_vax | -0.005 | 0.000 | -0.007 | -0.003 |
|  | outcome = log_ttd_est2_ |  |  |  |  |
| SM8 | intercept | 2.395 | 0.000 | 1.763 | 3.027 |
|  | dist | -0.150 | 0.000 | -0.184 | -0.116 |
|  | rod | -0.392 | 0.000 | -0.584 | -0.200 |
|  | em_cases | 0.044 | 0.000 | 0.023 | 0.065 |
|  | em_vax | -0.018 | 0.001 | -0.028 | -0.008 |
|  | tp_vax | 0.018 | 0.011 | 0.004 | 0.032 |
|  | outcome = log_ttd_obs2_ | | | | |
|  | The final Hessian matrix was not positive definite, although all convergence criteria were met. The MIXED procedure was continued and the validity of the results above cannot be guaranteed. | | | | |

Supplementary material Table S3. Additional ttd_est_ or ttd_obs_ models.

| model | covariate | reg. coefficient | p | 95% CI | |
| --- | --- | --- | --- | --- | --- |
|  |  |  |  | lower | upper |
| SM9 | intercept | 3.725 | 0.000 | 2.765 | 4.685 |
|  | dist | -0.067 | 0.000 | -0.081 | -0.053 |
|  | rod | 0.156 | 0.033 | 0.015 | 0.298 |
|  | tp_seq | 0.293 | 0.669 | -1.099 | 1.685 |
|  | vax_ratio_em | 0.970 | 0.311 | -0.964 | 2.904 |
|  | vax_ratio_tp | -2.101 | 0.005 | -3.494 | -0.708 |
|  | outcome = log_ttd_obs1_ | | | | |
| SM10 | intercept | 4.153 | 0.000 | 3.003 | 5.303 |
|  | dist | -0.100 | 0.000 | -0.125 | -0.075 |
|  | rod | -0.086 | 0.254 | -0.244 | 0.072 |
|  | vax_ratio_em | 0.393 | 0.710 | -1.874 | 2.660 |
|  | vax_ratio_tp | -2.081 | 0.010 | -3.568 | -0.593 |
|  | outcome = log_ttd_obs2_ | | | | |
| SM11 | constant | 3.661 | 0.000 | 3.400 | 3.922 |
|  | rod | 0.065 | 0.017 | 0.012 | 0.118 |
|  | tp_seq | -0.132 | 0.472 | -0.499 | 0.235 |
|  | vax_ratio_em | -0.299 | 0.188 | -0.750 | 0.152 |
|  | vax_ratio_tp | -0.444 | 0.017 | -0.805 | -0.083 |
|  | outcome = log_ttd_est1_ | | | | |

Supplementary material Table S4. Additional ttd_est_ models with third dose only variables (mostly equivalent to 1^st^ booster dose).

| model | covariate | reg. coefficient | p | 95% CI | |
| --- | --- | --- | --- | --- | --- |
|  |  |  |  | lower | upper |
| SM12 | constant | 3.407 | 0.000 | 3.258 | 3.556 |
|  | rod | -0.001 | 0.959 | -0.052 | 0.050 |
|  | tp_seq | -0.378 | 0.159 | -0.915 | 0.160 |
|  | boost_1_ratio_em | -0.030 | 0.569 | -0.134 | 0.075 |
|  | boost_1_ratio_tp | -0.563 | 0.000 | -0.707 | -0.418 |
|  | outcome = log_ttd_est1_ | | | | |
| SM13 | constant | 3.534 | 0.000 | 3.325 | 3.743 |
|  | rod | 0.022 | 0.376 | -0.029 | 0.074 |
|  | tp_seq | -0.271 | 0.240 | -0.735 | 0.193 |
|  | vax_ratio_em | -0.251 | 0.111 | -0.566 | 0.064 |
|  | boost_1_ratio_tp | -0.430 | 0.001 | -0.658 | -0.202 |
|  | outcome = log_ttd_est1_ | | | | |
| SM14 | constant | 3.348 | 0.000 | 3.175 | 3.521 |
|  | rod | -0.056 | 0.058 | -0.114 | 0.002 |
|  | tp_seq | -0.887 | 0.026 | -1.653 | -0.121 |
|  | boost_1_ratio_em | -0.048 | 0.477 | -0.185 | 0.089 |
|  | boost_1_ratio_tp | -0.538 | 0.000 | -0.679 | -0.398 |
|  | outcome = log_ttd_est2_ | | | | |
| SM15 | constant | 3.404 | 0.000 | 3.213 | 3.595 |
|  | rod | -0.049 | 0.093 | -0.107 | 0.009 |
|  | tp_seq | -0.773 | 0.037 | -1.494 | -0.051 |
|  | vax_ratio_em | -0.176 | 0.198 | -0.450 | 0.098 |
|  | boost_1_ratio_tp | -0.448 | 0.000 | -0.662 | -0.233 |
|  | outcome = log_ttd_est2_ | | | | |

Supplementary material Table S5. Covariance parameter statistics of the main models of this work.

| model | covariate | covariance parameter | p | 95% CI | |
| --- | --- | --- | --- | --- | --- |
|  |  |  |  | lower | upper |
| M1 | displacement 1 | 0.002 | 0.024 | 0.001 | 0.006 |
|  | displacement 2 | 0.002 | 0.054 | 0.001 | 0.007 |
|  | displacement 3 | 0.001 | 0.069 | 0.000 | 0.002 |
|  | intercept (subjects) | 0.000 | 0.761 | 0.000 | 0.057 |
| M2 | displacement 1 | 0.299 | 0.006 | 0.147 | 0.607 |
|  | displacement 2 | 0.001 | 0.035 | 0.000 | 0.003 |
|  | displacement 3 | 0.000 | 0.684 | 0.000 | 0.015 |
|  | intercept (subjects) | 0.001 | 0.121 | 0.000 | 0.002 |

Supplementary material Table S6. Discotope 3.0 calibrated scores for receptor binding domain (RBD) residues of SARS-CoV-2 S-protein, using predicted InterProIPRO18548 RBD domains. The higher the calibrated score, the higher the antigenicity of a given residue.

| InterPro (IPRO18548) definitions | variant / entity | | PDB structures | n (residues) | arithmetic mean | sd |
| --- | --- | --- | --- | --- | --- | --- |
|  | WT | 9 | | 5441 | 0.441 | 1.323 |
|  | delta | 11 | | 5898 | 0.468 | 1.277 |
|  | omicron | 10 | | 5811 | 0.361 | 1.221 |
|  | BA.1 | 11 | | 5570 | 0.339 | 1.264 |

Student’s t-test for comparison between entities: WT-delta p = 0.268, WT-BA.1 p < 0.001, delta-BA.1 p < 0.001, WT-omicron p < 0.001, delta-omicron p < 0.001.

Supplementary material Fig. S1. Example of classifying a strain into cluster, supercluster and variant (here shown for B.1. 529.2.75.3).


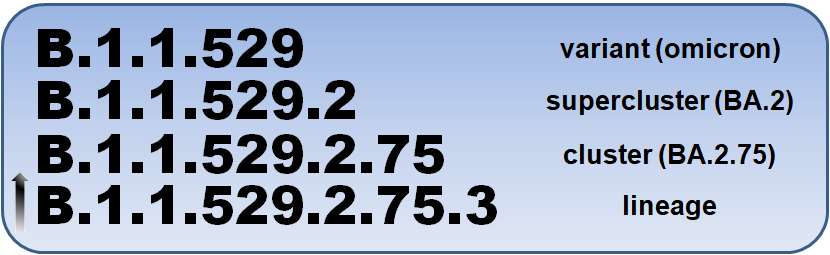


Supplementary material Fig. S2. Excerpt (screenshot from the outbreak.info platform[2]), indicating evidence for isolates allocated to the BA.1 supercluster (and thus to Omicron) as early as 2020. The screenshot was taken on 11 April 2024.


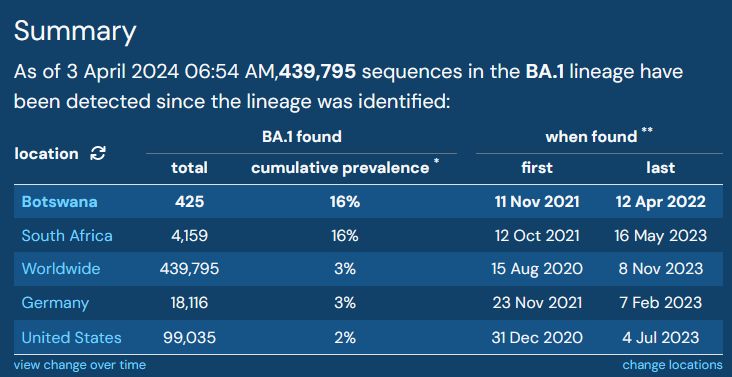


Supplementary material Fig. S3. Linear relationship assessment. Left panel: between tp_vax and em_vax. Right panel: between vax_ratio_tp and vax_ratio_em. A stronger linear association was apparent in the left panel.


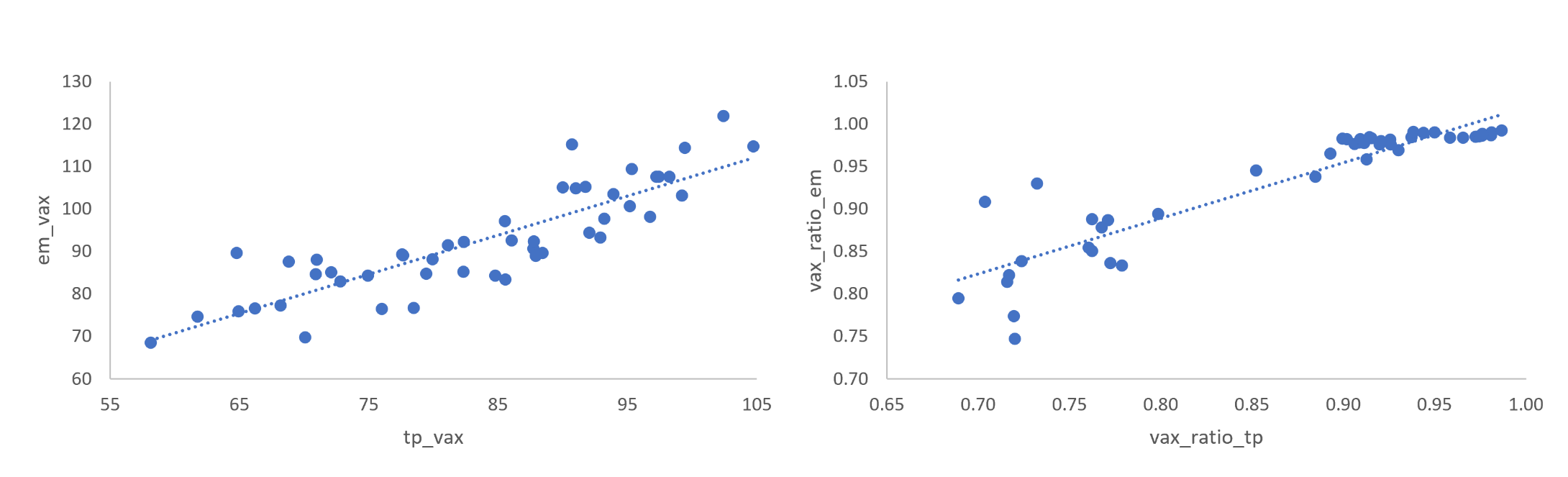

Supplement: Supplementary file 1 — Supplementary Material 1 [file 15010_2025_2586_MOESM1_ESM.docx]
